# Adherence to self-care behaviours and associated barriers in type 2 diabetes patients of low-and middle-income countries: a systematic review protocol

**DOI:** 10.1186/s13643-017-0436-4

**Published:** 2017-02-27

**Authors:** Victor Mogre, Natalie A. Johnson, Flora Tzelepis, Jonathan Shaw, Christine Paul

**Affiliations:** 1 School of Medicine and Public Health, University of Newcastle, University Drive, Callaghan, New South Wales 2308 Australia; 2 Department of Health Professions Education, School of Medicine and Health Sciences, University for Development Studies, P. O. Box TL 1883, Tamale, Ghana; 3 Hunter Medical Research Institute, Locked bag 1000, New Lambton, New South Wales 2305 Australia; 4 Hunter New England Population Health, Hunter New England Local Health District, Locked Mail Bag 10, Wallsend, New South Wales 2287 Australia; 5 Baker IDI Heart and Diabetes Institute, Melbourne, Victoria 3004 Australia

**Keywords:** Adherence, Barriers, Self-care, Type 2 diabetes, Low- and middle-income countries

## Abstract

**Background:**

Diabetes has become a global health emergency affecting high-, middle- and low-income countries. Previous systematic reviews have either focused on patients’ adherence to diabetes self-care behaviours only or barriers to diabetes care (including self-care) only in the published literature and have not also analysed data separately for low- and middle-income countries (LMICs). Thus, none have focused on adherence with, and barriers to, self-care behaviours from the perspectives of both patient and providers in low- and middle-income countries (LMICs). This systematic review will evaluate the published literature on adherence to five diabetes self-care behaviours (i.e., diet, exercise, self-monitoring of blood glucose, medication taking and foot care) and associated barriers in type 2 diabetes patients in LMICs. Healthcare providers’ barriers to the provision of diabetes self-care support will also be reviewed.

**Methods:**

This narrative review will be reported in accordance with the guidelines of the Preferred Reporting Items for Systematic review and Meta-Analysis Protocols (PRISMA-P). The electronic databases, MEDLINE, EMBASE, CINAHL, SCOPUS, PsycINFO, Cochrane Library and the British Nursing Index will be searched. Qualitative and quantitative studies reporting on type 2 diabetes patients’ adherence to self-care behaviours and associated barriers in LMICs will be included. Studies also reporting on barriers encountered by providers in LMICs providing diabetes care and supporting patients to adhere to self-care behaviours will also be included. Cross-sectional studies, observational cohort studies, baseline data of randomised controlled trials and qualitative studies will be eligible. Two independent reviewers will screen articles for inclusion, undertake quality assessment of included studies and execute data extraction using standardised forms. Discrepancies will be discussed to reach consensus, and another reviewer will adjudicate if the need arises. The Guidance of Narrative Synthesis in Systematic Reviews will be employed to explore relationships within and between included studies.

**Discussion:**

This review will provide evidence on adherence to self-care behaviours by type 2 diabetes patients in LMICs. Barriers experienced by patients in LMICs to adhere to recommended self-care behaviours will also be identified. Barriers experienced by healthcare providers in LMICs in providing self-care support patients will also be determined.

**Systematic review registration:**

PROSPERO CRD42016035406

**Electronic supplementary material:**

The online version of this article (doi:10.1186/s13643-017-0436-4) contains supplementary material, which is available to authorized users.

## Background

Diabetes affected 422 million adults in 2014 compared to 108 million in 1980 [1]. The increasing prevalence is associated with an increase in the prevalence of risk factors such as excess body weight, sedentary lifestyle, diet and ageing populations [2, 3]. In the last 10 years, the prevalence of diabetes has risen faster in low- and middle-income countries than in high-income countries [1]. The World Health Organization (WHO) anticipates that infectious diseases, maternal and infant mortality and malnutrition will be overtaken by non-communicable diseases such as diabetes mellitus (DM) in low- and middle-income countries (LMICs) by 2030 [4, 5]. Characterised by hyperglycaemia, insulin resistance and relative insulin deficiency, type 2 DM is the most frequently occurring form of DM globally [6–8].

Diabetes patients of LMICs are at increased risk of late diagnosis, poor diabetes control, hospitalisations and the development of diabetes-related complications [9, 10]. Also a higher proportion of premature deaths due to high blood glucose occurred in LMICs than in high-income countries [1].

Being a complex chronic disorder, diabetes care requires regular attention to diet, physical activity, monitoring of blood sugar, medications and foot care to attain positive health outcomes [11]. As has been advocated by clinical practice guidelines from the USA, UK and globally [12–15], these activities are referred to as diabetes self-care behaviours requiring the active participation of the patient in his or her care [15]. As each of these behaviours is important to health outcomes, it is essential to have a clear understanding of the level of adherence to each self-care behaviour and barriers to the performance of each of them for LMICs.

A number of reviews have examined adherence to self-care behaviours among type 2 diabetes patients [15] and barriers to diabetes care [16–18] including self-care [19]. The review by Coyle et al. included studies from low- and middle-income countries (LMICs) but only evaluated patients’ adherence to self-care behaviours (i.e. did not report on patients’ barriers to self-care) and did not analyse data from LMICs separately. The reviews by Pun et al. and Nam et al. [16–18] also included studies from LMICs, examined patient and provider barriers to general diabetes care including self-care but did not consider barriers to self-care behaviours separately and did not analyse LMICs data separately. Also, they did not undertake quality assessment of the included studies. The review by Sohal et al. evaluated patient and provider barriers and facilitators to diabetes management using only studies conducted in South Asia [18]. Wilkinson et al. [19] reviewed the literature to identify factors that influence patients’ ability to self-care for their diabetes, but only included qualitative studies, considered both type 1 and type 2 diabetes patients, did not include studies among providers and did not analyse LMICs data separately. Accordingly, a systematic review of the published literature from LMICs on patient adherence with, and the barriers to, self-care behaviours from the perspectives of both the patient and providers is warranted.

This systematic review will examine both qualitative and quantitative studies conducted in LMICs related to:

- Type 2 diabetes patients’ adherence to five self-care behaviours: recommended diet, sufficient exercise, diabetes medications, self-monitoring of blood glucose and foot care.
- Barriers to self-care behaviours among patients with type 2 diabetes
- Barriers to the provision of appropriate care to patients with type 2 diabetes among healthcare providers.

## Methods

This systematic review will be reported in accordance with the guidelines of the Preferred Reporting Items for Systematic review and Meta-Analysis Protocols (PRISMA-P) 2015 [20]. Details of this can be found in an additional file (see Additional file 1).

### Definition of low- and middle-income countries

Based on the 2015 Gross National Income per capita (GNI), low-income countries are those with a GNI per capita of ≤US$1,045 and middle-income countries are those with a GNI per capita of >US$1,045 but <US$12,736 [21]. Any country listed by World Bank as a LMIC at any point during the review period will be included.

### Information sources

Electronic databases including MEDLINE (OVID interface, 1948 and beyond), EMBASE (OVID interface, 1980 onwards), CINAHL, SCOPUS, PsycINFO, Cochrane Library and the British Nursing Index will be searched. The websites of relevant organisations such as the International Diabetes Federation (IDF), WHO and other diabetes-related institutions will also be examined for other potentially relevant studies. To ensure relevant publications are captured, the reference lists of relevant studies or systematic reviews will be hand searched.

### Search strategy

The databases will be searched using medical subject headings (MeSH) and free text words relating to the themes of this review (Table 1). Truncation and appropriate Boolean operators will be incorporated into the search strategy to cater for the different use of terms. Limitations pertaining to the inclusion and exclusion criteria will be set. An Ovid MEDLINE strategy will be developed by VM and other members of the research team under the guidance of a librarian with experience in systematic review searching. The finalized Ovid MEDLINE strategy will be adapted to the syntax and subject headings of the other databases. The search strategy will be reviewed by another librarian in accordance with the Peer Review of Electronic Search Strategies (PRESS) checklist. The execution of the search strategy will be done by VM.

**Table 1:** Search strategy

Searches	
1	diabetes mellitus.mp. or Diabetes Mellitus/
2	Diabetes Mellitus, Type 2/or Diabetes Mellitus, or diabetes mellitus type 2.mp.
3	(non insulin*dep* or noninsulin*dep*).mp. [mp = title, abstract, original title, name of substance word, subject heading word, keyword heading word, protocol supplementary concept word, rare disease supplementary concept word, unique identifier]
4	1 or 2 or 3
5	self-care.mp. or Self Care/
6	self-manage*.mp. or Patient Education as Topic/
7	self-care behaviours.mp.
8	5 or 6 or 7
9	diet.mp. or Diet/
10	Food Habits/or dietary habit*.mp.
11	nutrition.mp.
12	9 or 10 or 11
13	exercise.mp. or Exercise/
14	physical activity.mp.
15	13 or 14
16	Blood Glucose Self-Monitoring/
17	Self Care/or blood glucose testing.mp.
18	16 or 17
19	Self Care/or foot care.mp.
20	Patient Compliance/or medication taking.mp. or Self Administration/or Medication Adherence/
21	adherence.mp.
22	20 or 21
23	barrier.mp.
24	Health Services Accessibility/or Health Personnel/or patient barrier.mp. or “Attitude of Health Personnel”/
25	23 or 24
26	Developing Countries/or Socioeconomic Factors/or Low-income countries.mp.
27	Developing Countries/or middle-income countries.mp.
28	26 or 27
29	4 or 8 or 12 or 15 or 18 or 19 or 22 or 28
30	25 and 29
31	limit to (english language and yr = “1990 -Current” and “all adult (19 plus years)”)

*Represents Truncation

### Eligibility criteria

Studies will be included in accordance with the criteria outlined below.

- *Type of data*: Studies using quantitative, qualitative or both approach(es) will be included. Included studies will be limited to published papers including published theses/dissertations.
- *Study design*: Cross-sectional studies, cohort studies, baseline data from randomised controlled trials (RCTs) and qualitative studies (e.g. in depth interviews, focus groups) will be eligible for inclusion.
- *Study participants*: (i) Adults with type 2 diabetes from LMICs. Studies where the majority (≥50%) of participants are from a LMIC will be included. (ii) Studies that report on the barriers faced by healthcare professionals who deliver care to type 2 diabetes patients in LMICs will also be eligible for inclusion.
- *Search limitations*: All studies published from January 1990 onwards will be included. This time frame is informed by global estimates of diabetes that recognised diabetes as a global disease affecting adult populations of developing countries or LMICs [22].
- *Study focus*: Studies that are concerned with patient adherence to self-care behaviours and barriers to self-care. Also, studies that report on barriers experienced by providers to support patients to self-care for their diabetes will be included.
- *Setting*: Both population- and clinic-based studies will be eligible for inclusion.
- *Language*: Due to funding constraints, only studies published in English will be eligible for inclusion

### Exclusion criteria

Studies will be excluded if the majority of participants were younger than 18 years or had gestational diabetes. Conference proceedings, non-peer reviewed papers, opinion pieces, commentaries, case reports, abstracts and systematic reviews will be excluded.

### Data management, screening and selection

All search results will be downloaded into the reference manager ENDNOTE version X7 for screening. The liberal accelerated screening method for the screening of the literature search results will be adopted [23]. Firstly, VM will screen the title, abstract and subject headings of the citations in accordance with the eligibility criteria. Those that meet the eligibility criteria will be included and moved into the next level of assessment as well as those that were unclear. Those that are excluded as per the eligibility criteria will be reviewed by another member of the review team to confirm exclusion or otherwise. Secondly, full-text screening will be carried out by two members of the review team independently. Discussions and consensus building between the two reviewers will be used to resolve discordance and one or two arbitrators will adjudicate unresolved disagreements. In accordance with the PRISMA flow diagram, the entire selection process and reasons for exclusion will be recorded [24, 25]. Questions for screening will be developed in accordance with the eligibility criteria for the two-stage assessment of potentially eligible studies. The screening questions will be piloted, prior to the formal screening processes.

### Characteristics of included studies

The content of each included study will be extracted by two independent reviewers and recorded on a standard data collection form utilised in other reviews [24]. The following data will be extracted:

- *Publication details*: author(s) names, year of publication, country of study and years data were collected
- *Study setting*: population based, clinic-based
- *Study design*: cross-sectional, cohort, baseline data from RCT, qualitative (focus groups, in-depth interviews)
- *Details of study population*: demographics, sample size, eligibility criteria
- *Sampling methods*: consent rates
- *Measures*
- *Results*

During the data extraction process, disagreements will be resolved through discussion and consensus building and one or two arbitrators will adjudicate if needed.

### Quality assessment

All quantitative studies will be assessed using the National Heart, Lung and Blood Institute (NHLBI) standardised quality rating tools for quality assessment [26]. The NHLBI standardised tools are study-design specific [26, 27]. Studies having observational cohort and cross-sectional designs will be assessed using the NHLBI standardized Quality Assessment Tool for Observational Cohort and Cross-sectional studies. Data only from the baseline phase of RCTs will be included in this review because adherence to self-care behaviours and reporting of barriers may change at follow-up as a result of an intervention. Baseline data only from an RCT represents a cross-sectional assessment and therefore the NHLBI standardized Quality Assessment Tool for Observational Cohort and Cross-sectional studies will also be used for such studies.

The NLBI tools will allow for assessment of potential flaws in study methods including sources of bias, sampling, confounding, study power and other relevant factors. Each study will be judged as “good”, “fair” or “poor” quality based on ratings of a list of items included in the tools. Globally, a good study has the least risk of bias and is considered valid. A fair study is prone to some bias but insufficient to invalidate its findings, varying in its strengths and weaknesses. A poor study has high risk of bias and is considered invalid. Quality assessment will be conducted independently by two members of the review team. Differences will be resolved through discussions between these two and if unresolved the other members of the team will be consulted for adjudication.

Qualitative studies will be assessed according to quality assessment categories adopted by Popay, Rogers and Williams [28]. These categories will pertain to relevance and appropriateness of research design, study context, use of convenience/purposive sampling, richness of data, thoroughness of data analysis and clarity of interpretation and logical generalisation of findings based on theoretical underpinnings. This tool has been used widely for assessing the quality of qualitative studies in several systematic reviews [29–31]. It has been reported to be applicable across a wide range of qualitative methods [30, 32].

### Data analysis and synthesis

A systematic narrative synthesis will be conducted. Tables and narrative summaries will be used to present the characteristics and findings of included studies. Relationships within and between included studies will be explored as well as assessment of the robustness of the synthesis, in accordance with the Guidance of Narrative Synthesis in Systematic reviews produced by the Economic and Social Research Council (ESRC) methods programme [33]. This guidance provides a framework for the execution of narrative synthesis using both broad and specific techniques and tools. It has been used widely in several systematic reviews [34–38]. VM will conduct the synthesis in consultation with the rest of the review team members.

## Discussion

Systematically reviewing the published literature from LMICs will increase knowledge on the self-care behaviours that are commonly adhered to and those that are not among type 2 diabetes patients in LMICs. It will also provide data relating to barriers faced by patients to self-care for their diabetes and providers to support patients to self-care in LMICs which will assist with the design of interventions to improve care planning and delivery in this population. The inclusion of both qualitative and quantitative studies will allow for a comprehensive review of the published literature.

## Additional files

Additional file 1: PRISMA-P checklist. This checklist provides a list of recommended items to include in a systematic review protocol and the pages upon which those items are covered in the protocol. (DOCX 30 kb)

## Abbreviations

ESRC: Economic and social research council
GNI: Gross national income per capital
IDF: International diabetes federation
LMICs: Low- and middle-income countries
NHLBI: National Heart, Lung and Blood Institute
PRESS: Peer review of electronic search strategies
PRISMA-P: Preferred Reporting Items for Systematic Review and Meta-Analysis Protocol
RCTs: Randomised controlled trials
WHO: World Health Organization
